# The effectiveness of computerized cognitive training on cognitive functions and mental health in people with schizophrenia

**DOI:** 10.1192/j.eurpsy.2024.1494

**Published:** 2024-08-27

**Authors:** Y. Haghgoo, H. A. Haghgoo

**Affiliations:** ^1^University of Social Welfare and rehabilitation Sciences, Tehran, Iran, Islamic Republic Of; ^2^Occupational Therapy, University of Social Welfare and rehabilitation Sciences, Tehran, Iran, Islamic Republic Of

## Abstract

**Introduction:**

People with schizophrenia have multiple and persistent cognitive deficits. These defects have a deep impact on people’s psycho-social functions. Although computerized cognitive training has positive results in some people, the effect of these treatment programs in schizophrenia is not clear.

**Objectives:**

The purpose of this study was to investigate the effectiveness of computerized cognitive exercises on the components affecting the mental health and cognitive functions of schizophrenic patients.

**Methods:**

Fifty-four adults with schizophrenia were randomly divided into two intervention and control groups. Participants in the intervention group received 30 sessions of 5-45 minutes of computerized cognitive training in addition to the usual treatment programs. While the control group only received their usual rehabilitation programs (Pharmacotherapy, psychotherapy and occupational therapy).

Mental health was evaluated with Warwick-Edinburgh Mental Well-Being Scale and Depression-Anxiety-Stress Scale (DASS), and cognitive functions with CANTAB tests Batteries before the intervention, after and two months after the intervention (follow up). The set of CANTAB tests used in this study included the following tests: Spatial Recognition Memory (SRM), Paired Associates Learning (PAL), Stockings of Cambridge(SOC), Spatial Working Memory (SWM), and Spatial Span (SSP).

**Results:**

The analysis of the findings showed that the patients’ performance in the cognitive tests related to memory and executive functions improved significantly in the intervention group after the intervention. In problem solving skills, despite the better performance in the intervention group, the difference between the two groups was not significant. Also, the intervention was able to significantly improve mental health and reduce stress. But no significant difference was observed in reducing anxiety and depression.

**Conclusions:**

As a result, the study showed that 30 sessions of computerized cognitive training can have a positive effect on overall mental health and some cognitive functions.

**Disclosure of Interest:**

None Declared

